# Effect of Zinc on Humoral and Cell-Mediated Immunity of Broilers Vaccinated Against Coccidiosis

**Published:** 2013

**Authors:** Sobhan SAJADIFAR, Hadi MIRANZADEH, Milad MOAZENI

**Affiliations:** 1 Department of Epidemiology and Parasitology, Faculty of Veterinary Medicine and Zootechnics, Armenian National Agrarian University, Yerevan, Armenia; 2 Department of Veterinary, University of Applied Science and Technology, Institute of Science Applied Higher Education of Jahad -e- Agriculture, Isfahan, Iran

**Keywords:** Coccidiosis, Zinc, Immunity

## Abstract

**Background:**

The aim of the present study was the comparison of humoral and cell-mediated immunity in broilers fed with different levels of zinc during a coccidiosis challenge.

**Methods:**

One hundred and forty-four one-day-old broiler chicks were used with three dietary zinc (40, 120 and 200 mg/kg). At 14 d of age, all birds were inoculated orally with 5×10^3^ sporulated oocysts of *E. Tenella*. At 2, 22, 32, 42 days of age, the blood serums were tested for antibody titer against Newcastle disease vaccine, using the standard HI test. On day 42 the sum of nitrite and nitrate based on the reduction of nitrate to nitrite by cadmium and white blood cell count (WBC) using a hemocytometer were measured.

**Results:**

At 42 d, levels of 120 and 200 mg significantly (*P*< 0.05) increased the antibody titer in compare with the control. The peak response of CBH was observed at the level of 200 mg Zn/kg diet. Also both level of 120 and 200 mg Zn/kg diet increased WBC count and sum of nitrite and nitrate in serum compared with the control.

**Conclusion:**

The levels of 120 and 200 mg Zn/kg diet could be considered as a non-pharmacologic booster of immunity in broilers chicks infected with *E. Tenella*.

## Introduction

Coccidiosis is a parasitic disease that causes the enormous economic loss in poultry production. Williams (1) reported this economic loss is about 800 million $ annually around the world. The positive manipulation of humoral and cell-mediated immune responses would be considered as a proper way to restrain the infectious diseases. There is increasing evidence that the concentrations of trace elements required for healthy animals are often below what is required for animals experiencing an immunological challenge (2). Studies indicated that supplementing the diet of broilers with additional zinc was efficient to improve immunity against diseases. Consumption of zinc higher than requirement increased disease resistance (3). Zn had effect on cellular and humoral immunity (4), besides it had role in gene expression, mitosis, and apoptosis of lymphoid cells (3). In other hand although in large number of studies additional zinc used in diet of broilers has improved antibody production (5) but in some other studies this result has not been observed (6). The aim of the present study was the comparison of humoral and cell-mediated immunity in broilers fed with different levels of zinc during a coccidiosis challenge.

## Materials and Methods

### Animals and treatments

One hundred and forty-four one-day-old (Ross 308) broiler chicks were used in this experiment. The birds were randomly assigned to 6 treatment groups consisting of 4 replicates of 12 birds. The experimental diets were manufactured from a basal diet (Table 1), which was formulated using the National Research Council (7) Tables. Three Zn levels (40, 120 and 200mg Zn/kg diet) were added to the basal diet to establish the treatments. Zinc supplementation was provided by ZnSo_4_. On d 7, all broilers were vaccinated against coccidiosis (Livacox Q^®^, Merial) at a dose of 2mL/300mL of water distributed in 2mL for each broiler via oral administration. Zinc contents in starting and finishing basal diets and potable water were 72, 70 and 5 mg/kg respectively, as measured by atomic absorption analysis. Birds were kept in floor pens, and diets and fresh water were provided *ad libitum* from the first day.

**Table 1:** Ingredients and calculated composition of the starter and finisher diets

Ingredients	Starter (%)	Finisher (%)
Corn	53.55	**59.57**
Soybean meal 44%CP	38.93	**33.34**
Monodibasic Phosphate	1.43	**1.21**
Limestone	1.35	**1.38**
Vegetable oil	3.84	**3.51**
Salt	0.41	**0.43**
DL-methionine	0.207	**0.214**
L-Lysine HCl	0.129	**0.197**
Choline HCl 60%	0.06	**0.05**
Mineral-vitamin premix^1^	0.1	**0.1**
Total	100	**100**
Calculated Nutrients		
Crude protein %	22	**20**
ME, kcal/kg	3,050	**3,100**
Calcium, %	0.9	**0.85**
Available phosphorus, %	0.4	**0.35**
Sodium, %	0.2	**0.21**
Chloride, %	0.27	**0.29**
Digestible Lys, %	1.15	**1.07**
Digestible Met,, %	0.49	**0.48**
Digestible Met+Cys %	0.81	**0.77**
Digestible Thr, %	0.78	**0.71**
Choline, mg/kg	1,420	**1,300**

1 Composition (per kg): manganese, 75,000 mg; iron, 50,000 mg; copper, 8,000 mg; iodine, 750 mg; vitamin A, 8,000 kIU; vitamin D3, 2,000 kIU; vitamin K3, 1,800 mg; vitamin B1; 1,800 mg; vitamin B2, 6,000 mg; vitamin B6, 2,800 mg; vitamin B12, 12,000 µg; pantothenic acid, 10,000 mg; niacin, 40,000 mg; folic acid, 1,000 mg; biotin. 60,000 µg; selenium, 0.3 mg/kg. Basal diets Zn measured by atomic absorption spectrometer and Zinc contents were 74 and 72 mg/kg in starting and finishing basal diets.

### E. Tenella challenge

At 14 d of age, all birds were inoculated orally with 5×10^3^ sporulated oocysts of *E. Tenella* by potable water. Oocyst production and shedding were assessed as described by Lillehoj & Ruff (8).

### Antibody titer against Newcastle disease vaccine (NDV)

Birds of all groups were intramuscularly injected with 0.1 ml of killed NDV vaccine (Cevac^®^Broiler NDK) at eight days of age. Two blood samples from each replicate were collected at 2, 22, 32, 42 days of age. All the blood samples obtained from wing vein and serums were separated by 3000 rpm centrifuging for 15 min. The serums were tested for antibody against NDV, using the standard Haemagglutination Inhibition (HI) test (9) and the results were expressed as the log_2_.

### Cutaneous basophil hypersensitivity (CBH) response

At 28 days of age, 4 chicks from each treatment were color-marked and sensitized with 0.25mL of 2, 4-dinitrochlorobenzene (DNCB) solution (10 mg/mL asetone). DNCB was purchased from SIGMA Aldrich^®^ (2,4-Dinitrochlorobenzene, SKU 237329). The inoculation was made in the interdigital space between the third and fourth toes of right foot by intradermal injection. In the same interdigital space of the left foot (in the same bird), 0.25 mL acetone was injected as control. At 24, 48, 72 h post-DNCB challenge, the cell reaction caused by DNCB was evaluated as CBH response by measuring skin thickness with electronic caliper (Eletronic Digital Caliper, with 0.01mm precision).

### Measurements

The thickness of interdigital spaces was measured before the injection and 24, 48 and 72 hours afterwards using a digital caliper. The results were used to calculate the following:

1. Response = post-DNCB injection thickness of the right foot – pre-DNCB injection thickness of the right foot (mm)
2. Acetone control response = post-DNCB injection thickness of the right foot – pre-DNCB injection thickness of the left foot (mm)

Therefore, cell reaction at each evaluation time was calculated as: CBH = 1) – 2)

### Total white blood cells (WBC) counts

On 42d, blood samples were collected from wing vein using sterile lancet. Briefly, 490µ / of brilliant cresly blue dye was mixed with 10µ / whole blood sample and total leukocytes were counted using a hemocytometer.

### Determination of sum of nitrite and nitrate

Serum samples were prepared from eight chicks per each treatment at 42 d. Measurements of nitrite and nitrate was based on the reduction of nitrate to nitrite by cadmium. The nitrite produced was determined by Griess reaction. At this method, the serum sample was deproteinized by adding ZnSO (75 mmol/l) and NaOH (55 mmol/l) solutions. After centrifuging, the supernatant was recovered and diluted in glycine buffer (45 g/l, pH 9.7). Cadmium granules (2-2.5 g) were rinsed three times with deionized distilled water and swirled in a CuSO4 solution (5 mmol/l) in glycine-NaOH buffer (15 g/l, pH 9.7) for 5 min to become activated. Freshly activated cadmium granules were added to pretreated deproteinized serum. After continuous stirring for 10 min, the samples were transferred to appropriately labeled tubes for nitrite determination by Griess reaction. Griess reagent 1 (1% sulfanilamide in 5% phosphoric acid) was added to the sample tubes and then incubated for 10 minutes at room temperature, protected from light. Griess reagent 2 was added (0.1% N-napthylethylenediamine dihydrochloride in water) to all samples and absorbance was measured within 10 minutes in a spectrophotometer at a wavelength of 540 nm (10). At this stage of the experiment, the sum of the nitrite and nitrate was measured.

### Statistical analysis

Statistical analyses were conducted using the ANOVA general linear models procedure of SAS software (11). When ANOVA revealed significant effects, means were separated by Duncan's multiple range tests. The values were considered significant at *P*< 0.05.

## Results

**Fig. 1:**
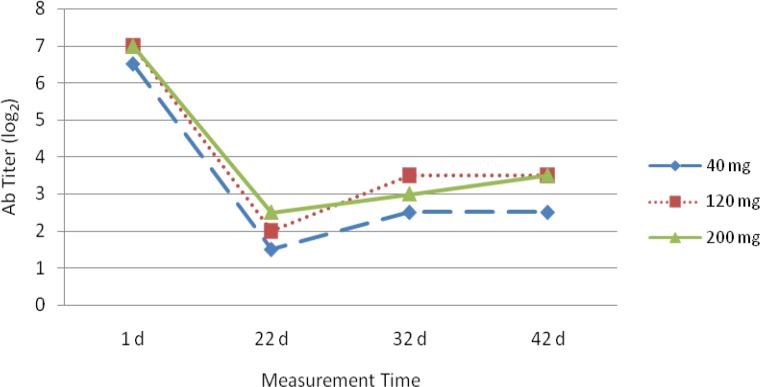
Antibody titer against NDV in blood of broilers fed different levels of zinc

Figure 1 shows the effects of different treatments on antibody titer against NDV. At the age of 22 and 32 d, birds receiving 200 and 120 mg zinc had a superior antibody respectively. At 42 d, although there was no significant difference between these two levels but both levels significantly (*P*< 0.05) increased the humoral immunity against NDV compared with the control.

**Fig. 2:**
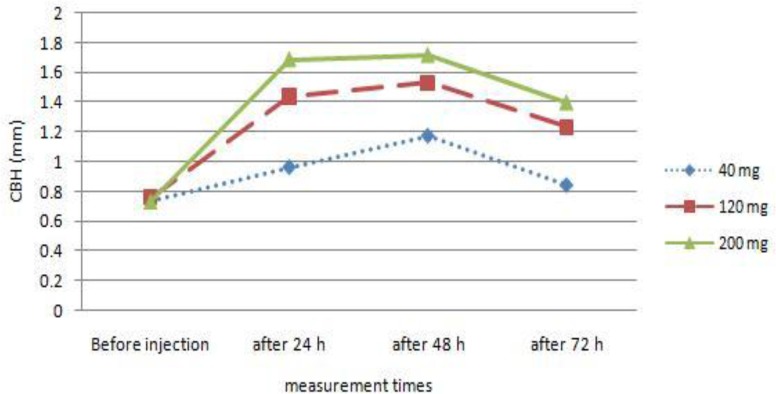
CBH response in broilers fed different levels of zinc

Also there was a significant difference (*P*< 0.05) in mean skin thicknesses among the groups. Figure 2 shows that skin thickness increased slowly, reaching its peak around 48h after DNCB challenge and attenuating subsequently. The peak response was observed at the 200 mg/kg level.

**Fig. 3:**
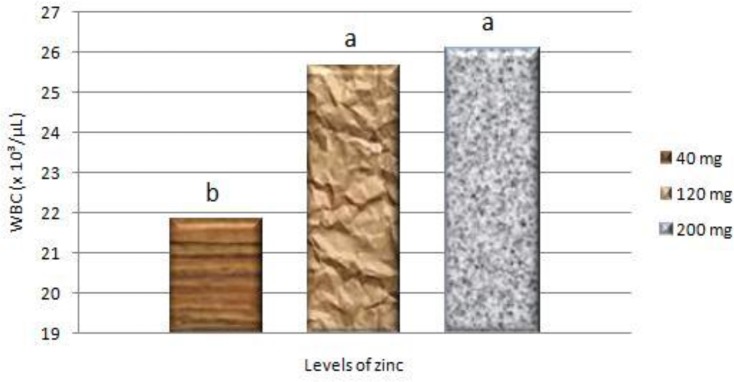
White blood cell (WBC) count of broilers fed different levels of zinc ^a,b^ Columns that do not share the same letters differ significantly (*P*< 0.05)

Figure 3 displays the effects of Zn supplementation on white blood cell (WBC) count. Levels of 120 and 200 mg Zn/kg diet were similar and significantly (*P*< 0.05) enhanced WBC count compared with 40 mg/kg level

**Fig. 4:**
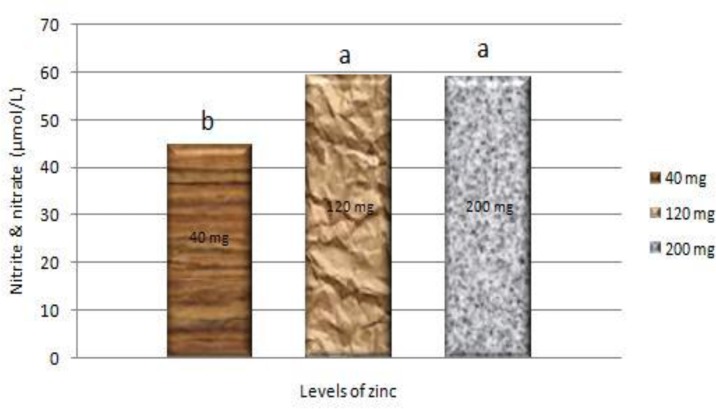
Sum nitrite and nitrate in serum of broilers fed different levels of zinc ^a,b^ Columns that do not share the same letters differ significantly (*P*< 0.05)

Figure 4 shows the effects of Zn supplementation on sum nitrite and nitrate in the serum. Although we did not observe any significant difference between the levels of 120 and 200 mg Zn/kg diet but both levels significantly (*P*< 0.05) increased the sum of nitrite and nitrate compared with the control group.

## Discussion

Humoral immunological response was improved with higher levels of zinc at 22, 32 and 42 days old by HI. Bartlett & Smith (6) reported the birds fed 68 and 181 mg zinc showed a higher IgM, and IgG antibodies. Also Kidd et al. (12) demonstrated that higher Zn levels produce better immune status in broiler chickens. Fraker et al. (13) concluded that “zinc is essential for thymulin, a thymic hormone that regulates T-lymphocyte maturation”. Nassiri-Moghadam & Jahanian (14) reported that “more zinc could enhance maturation of T-lymphocytes and activation of B-lymphocytes by inducing thymulin activity and therefore improve immunity in birds”.

Improved CBH response was observed with higher levels of zinc and the peak was at the 200 mg/kg level. These finding was in accordance with the observations of Sunder et al. (15) that the peak response was observed at the 160-ppm level of Znso_4_ in compare with 40 and 80-ppm levels. Kidd et al. (16) indicated that Zn improved the counts of thymocytes, peripheral T-cells and interferon. Increase of cell-mediated immunity in this work could be related to the production of interleukin-2, which was supported by higher Zn level in the diet (16) and effect of zinc on development of white blood cells into T-lymphocytes with specific functions like T helper and killer cells (17).

Broilers supplemented with 120 and 200 mg/kg of Zn in the diet showed improved WBC count than the control group. It was in agreement with the finding of Al-Daraji & Amen (18) that addition of zinc to the diet of broiler breeder chickens (75 and 100 mg/kg) resulted in increase in blood plasma protein in comparison with the control group. Whereas Hosseini et al. (19) reported no significant effect on WBC in broilers fed different levels of dietary Zn. Zinc is required for lymphocyte proliferation (20), monocyte (21) and neutrophils (22).

The sum of nitrite and nitrate in the serum was increased in groups fed with 120 and 200 mg Zn/kg diet. Increase in the sum of nitrite and nitrate is a result of increase in production of macrophage. Tizard (23) proposed “when exposed to antigens or chemotactic agents, macrophages will begin to produce iNOS. This enzyme leads to the production of nitric oxide, which will subsequently react with superoxide anions to generate toxic derivatives, allowing macrophages to proficiently kill several types of pathogens”. Zinc has effect on some aspects of macrophage function too. Shankar & Prasad (3) reported that “resistance to some diseases after supplementation with Zn, may be due to its role in nitric oxide–mediated microbicidal activity of macrophages”. Our result was in agreement with the finding of Bartlett & Smith (6) that “birds fed 181 mg Zn/kg diet had more activated macrophages for opsonized and unopsonized SRBC than those fed with the lower Zn”.

In general this study showed that supplementation of diet of broilers above 40 mg Zn/kg diet increased different aspects of immunity even in broilers vaccinated against coccidiosis. So that additional zinc improved WBC and macrophages activity which could be helpful in treatment of coccidiosis. Supplementation of diet with 120 and 200 mg Zn were similar in improving the antibody titer against NDV. However the level of 200 mg Zn/kg diet was more efficient to improve CBH response, but the level of 120 mg Zn/kg diet was enough to achieve the maximum WBC count and sum of nitrite and nitrate in serum.

## Conclusion

The overall results of our study illustrated that different parameters of immune system could be affected by using different levels of zinc in diet and both level of 120 and 200 mg Zn/kg diet, could be considered as a non-pharmacologic booster of immunity in broilers chicks infected with *E. Tenella* even after vaccination against coccidiosis.
